# Antibiotic Resistance in an Indian Rural Community: A ‘One-Health’ Observational Study on Commensal Coliform from Humans, Animals, and Water

**DOI:** 10.3390/ijerph14040386

**Published:** 2017-04-06

**Authors:** Manju Raj Purohit, Salesh Chandran, Harshada Shah, Vishal Diwan, Ashok J. Tamhankar, Cecilia Stålsby Lundborg

**Affiliations:** 1 Department of Public Health Sciences, Global Health—Health Systems and Policy (HSP): Medicines Focusing Antibiotics, Karolinska Institutet, 17177 Stockholm, Sweden; saleshp@gmail.com (S.C.); vishal.diwan@ki.se (V.D.); ejetee@gmail.com (A.J.T.); cecilia.stalsby.lundborg@ki.se (C.S.L.); 2 Department of Pathology, R.D. Gardi Medical College, Ujjain 456006, India; 3 Department of Microbiology, R.D. Gardi Medical College, Ujjain 456006, India; sharshada1955@yahoo.com; 4 International Centre for Health Research, Ujjain Charitable Trust Hospital and Research Centre, Ujjain 456006, India; 5 Department of Public Health and Environment, R.D. Gardi Medical College, Ujjain 456006, India; 6 Indian Initiative for Management of Antibiotic Resistance, Department of Environmental Medicine, R.D. Gardi Medical College, Ujjain 456006, India

**Keywords:** antibiotic resistance, community, environment, India, coliforms, commensal

## Abstract

Antibiotic-resistant bacteria are an escalating grim menace to global public health. Our aim is to phenotype and genotype antibiotic-resistant commensal *Escherichia coli (E. coli)* from humans, animals, and water from the same community with a ‘one-health’ approach. The samples were collected from a village belonging to demographic surveillance site of Ruxmaniben Deepchand (R.D.) Gardi Medical College Ujjain, Central India. Commensal coliforms from stool samples from children aged 1–3 years and their environment (animals, drinking water from children's households, common source- and waste-water) were studied for antibiotic susceptibility and plasmid-encoded resistance genes. *E. coli* isolates from human (*n* = 127), animal (*n* = 21), waste- (*n* = 12), source- (*n* = 10), and household drinking water (*n* = 122) carried 70%, 29%, 41%, 30%, and 30% multi-drug resistance, respectively. Extended spectrum beta-lactamase (ESBL) producers were 57% in human and 23% in environmental isolates. Co-resistance was frequent in penicillin, cephalosporin, and quinolone. Antibiotic-resistance genes *bla_CTX-M-9_* and *qnrS* were most frequent. Group D-type isolates with resistance genes were mainly from humans and wastewater. Colistin resistance, or the *mcr-1* gene, was not detected. The frequency of resistance, co-resistance, and resistant genes are high and similar in coliforms from humans and their environment. This emphasizes the need to mitigate antibiotic resistance with a ‘one-health’ approach.

## 1. Introduction

Antibiotic resistance represents a significant and complex global health problem. Global consumption of antibiotics has increased by nearly 40% in the last decade [1]. Apart from fundamental applications in clinical settings, very large amounts of antibiotics are used in agriculture, the food industry, and aquaculture [2]. Due to incomplete metabolism and the environmental spread of unused antibiotics, they enter the ecosystem, serving as a potent stimulus to elicit a bacterial adaptation response to develop antibiotic resistance and genes [3,4]. The accumulation of antibiotics in the environment facilitates the spread of antibiotic resistance genes. Various resistance mechanisms are continuously emerging and spreading globally, which threatens our ability to treat common infectious diseases, resulting in increased death, disability, and costs. TheWorld Health Assembly, in 2015, thus adopted a global action plan on antimicrobial resistance focussing on bacterial resistance [5].

There is a worldwide concern about the emergence of antibiotic resistance in bacteria carried by healthy individuals, so-called commensal bacteria. Commensal bacteria from the gut microbes, e.g., coliforms, may play a crucial role in the spread of resistance within a community. Surveillance data shows that resistance in *Escherichia coli* is generally consistently highest for antimicrobial agents that have been in use the longest time in human and veterinary medicine [6]. *E. coli* is also considered an indicator bacteria of antibiotic resistance. Animal and human fecal flora and the environment, including water sources, serve as natural habitats and reservoirs of antibiotic-resistant bacteria and resistance genes. Antibiotic resistance in wastewater, surface water, and drinking water is well documented [7,8].

Thus, within the community, resistant bacteria circulated from person to person or from animals and environment to person, or vice versa. The epidemiology of antibiotic-resistant microorganisms at the human-animal-environmental interface involves complex and largely unpredictable systems that include transmission routes of resistant bacteria, as well as resistance genes and the impact of antibiotic-selective pressures in various reservoirs (animals, humans, and the environment). Though the presence and patterns of antibiotic resistant commensal indicator bacteria *E. coli* isolates from humans, animals, and water have been studied in isolation, it is now recognized that they need to be studied together, i.e., using the ‘one-health’ approach [5]. Thus, our aim is to determine and compare the antibiotic resistance pattern among commensal coliforms and *E. coli* from humans, animals, and water from the same community.

## 2. Materials and Methods

### 2.1. Study Setting and Sample Collection

**Figure 1:**
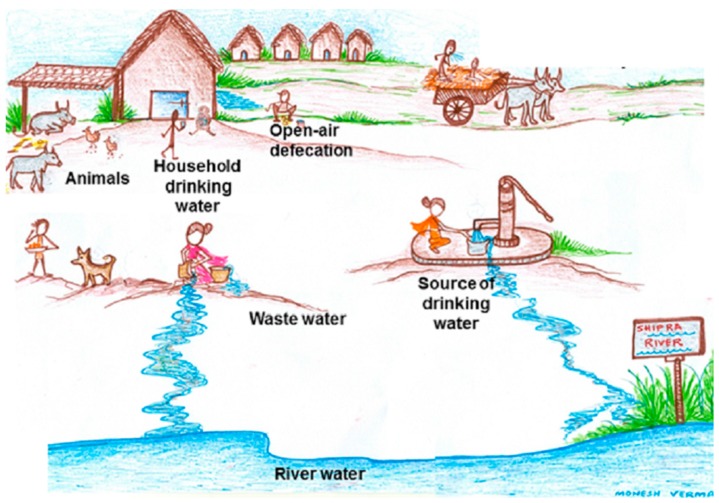
“One-health” approach.

The present study is a part of an ongoing project that has been described in detail previously [9]. In brief, the study was conducted in Ujjain district of Madhya Pradesh, India. We selected the village from the demographic surveillance site of Ruxmaniben Deepchand (R.D.) Gardi Medical College having poor literacy and living standards (Table 1) as described in [9]. The children aged between 1 and 3 years in the village at the commencement of the study, i.e., September 2014 were identified. Trained research assistants visited selected children’s homes and informed the children’s parents/guardians about the study. All children whose parents consented for their children to participate were included in the study. Stool samples from selected children and drinking water samples from their households were collected. Stool samples from five different animals (cattle, hen, dog, goat, and horse), which commonly share their environment with children, two common drinking-water sources, and two waste-water samples from the village were also collected, as depicted in Figure 1. All of the collected samples were transported within five hours to the Central Research Laboratory at R.D. Gardi Medical College. All samples could be collected within three days from the village. The village health worker in a predesigned format noted basic socio-demographic details by interviewing the head of the family.

### 2.2. Identification of Coliforms and Confirmation of E. coli

Microbiological processing of the samples was started as soon as the samples were received in the laboratory. The samples were processed on selective and differential HiCrome coliform chromogenic agar (HiMedia Laboratories Pvt. Ltd., Mumbai, India) to identify *E. coli* (blue-violet colony) and non-*E. coli* coliforms (*Citrobacter freundii* and *Enterobacter cloacae*—salmon to red, *Klebsiella pneumoniae*—light pink, *Salmonella enteritidis* and *Shigella flexneri*—colorless) as described in detail [9]. The presumptive *E. coli* were confirmed by PCR (mentioned in detail below). Briefly, stool samples were inoculated at 37 °C for 24 h directly on the chromogenic agar while the water samples were first filtered through membranes [9], followed by inoculation of the membranes on agar plate. In water samples, colony-forming units (CFUs) per unit volume of sample were estimated for total coliforms and *E. coli* to provide a snapshot of the abundance of coliforms and the *E. coli* load in the samples tested. Six *E. coli* and two colonies from every type of non-*E. coli* were isolated, purified, stored, and processed for antibiotic susceptibility testing and DNA extraction.

### 2.3. Antibiotic Susceptibility Testing

All the pure and confirmed six *E. coli* and HiCrome coliform agar categorized non-*E. coli* isolates from each sample were analyzed for the susceptibility to colistin, ampicillin, ceftriaxone, cefepime, ciprofloxacin, tetracycline, tigecycline, meropenem, imipenem, gentamicin, amikacin, sulphamethoxazole, cotrimoxazole, nalidixic acid, and nitrofurantoin (all purchased from HiMedia Laboratories Pvt. Ltd., Mumbai, India) by the Kirby-Bauer disc diffusion method as described in [9]. The results of inhibitory zones of the antibiotic susceptibility testing procedure were interpreted as detailed previously [9] using the Clinical and Laboratory Standards Institute (CLSI) criteria. The isolates were categorized as the number of resistant isolates per antibiotic type per sample (out of six isolates), phenotypically-confirmed beta-lactamase producers (only where beta-lactamase production is indicated as a possible mechanism explaining observed resistance) by the combined disc diffusion method(isolates resistant to either ceftazidime (HiMedia Laboratories Pvt. Ltd., Mumbai, India) or ceftriaxone (third generation cephalosporin), the presence of co-resistance (phenotypic resistance to two or more antibiotics of same or different group per isolate), and multidrug resistance (MDR) (MDR co-resistance involving three or more antibiotics of three different groups) in each sample. *E. coli* reference strain ATCC 25922 was used for quality control. Intermediate resistant isolates were categorized as resistant.

### 2.4. Amplification of Genes

The total bacterial DNA from *E. coli* isolates was extracted using the alkaline lysis method [10]. The genetic confirmation of *E. coli* was done through PCR with genus-specific oligonucleotide primers [11]. β-lactamase-encoding (*bla*_C*TXM*_, *bla_TEM_*, and *bla_SHV_*); plasmid-mediated quinolone resistance (*qnrA*, *qnrS*, *qnrS*, *aac(6′)-Ib-cr*, and *qepA*), carbapenem resistant (*VIM*, *NDM*, *IMP*) and colistin resistant (*mcr-1*) genes were amplified and identified with previously-described primers [12,13] for all *E. coli* isolates. The phylogenetic grouping of all *E. coli* was performed based on *chuA*, *vjaA*, and *TspE4C2* genes which were amplified by multiplex PCR as described in detail elsewhere [14]. All of the amplified PCR products were visualized using a gel documentation system for all *E. coli* isolates.

### 2.5. Data Analysis

Drug susceptibility and gene detection data were generated, and entered into IBM SPSS Statistics 23.0 (SPSS Inc., Chicago, IL, USA). Descriptive statistics, frequencies, and bivariate analyses (cross-tabulations) for the susceptibility pattern of *E. coli* isolates from different samples were calculated. Multiple linear regression analysis was performed to assess the effect of demographic features on antibiotic resistance. Resistance to different antibiotics was included as dependent variables, and age, sex, and other demographic parameters were included as independent variables in the model to adjust for confounding variables. Differences were considered statistically significant at *p* < 0.05. Results were also noted for the variation in coliforms load (in terms of CFU per 100 mL) in drinking water and wastewater. The results of the susceptibility pattern of *E. coli* and non-*E. coli* were correlated with the corresponding pattern and between human and environmental samples.

### 2.6. Ethical Consideration

Ethical issues: Ethics permission for the study was obtained from the Institutional Ethics Committee of R.D. Gardi Medical College, Ujjain (India) (No. 2013/07/17-311). Parents/guardians were explained about the purpose of the study, about voluntary participation, and were assured by researchers to maintain confidentiality. Oral and written informed consent was taken, thereafter. Children identified as having need of medical care were referred and treated at the Department of Pediatrics at C.R. Gardi Hospital.

## 3. Results

### 3.1. Study Samples

A total of 24 children were identified according to the inclusion criterion from the selected village. Stool samples from 22 children and drinking water from their respective home were collected. Samples from two children (one not at home, one did not passed motion by the time of collection) could not be collected even after two follow-up visits. Table 1 shows the demographic details of the families of the children from whom isolates were obtained. All of the isolates identified as blue-violet colonies on HiCrome agar were confirmed by PCR as *E. coli* while other bacterial isolates identified on HiCrome were considered together as the non-*E. coli* group and processed for antibiotic susceptibility testing.

The number and source of the samples and the number and types of coliforms isolated and studied from each sample is shown in Table 2.

### 3.2. Antibiotic Resistance Pattern of E. coli in Various Sources

**Figure 2:**
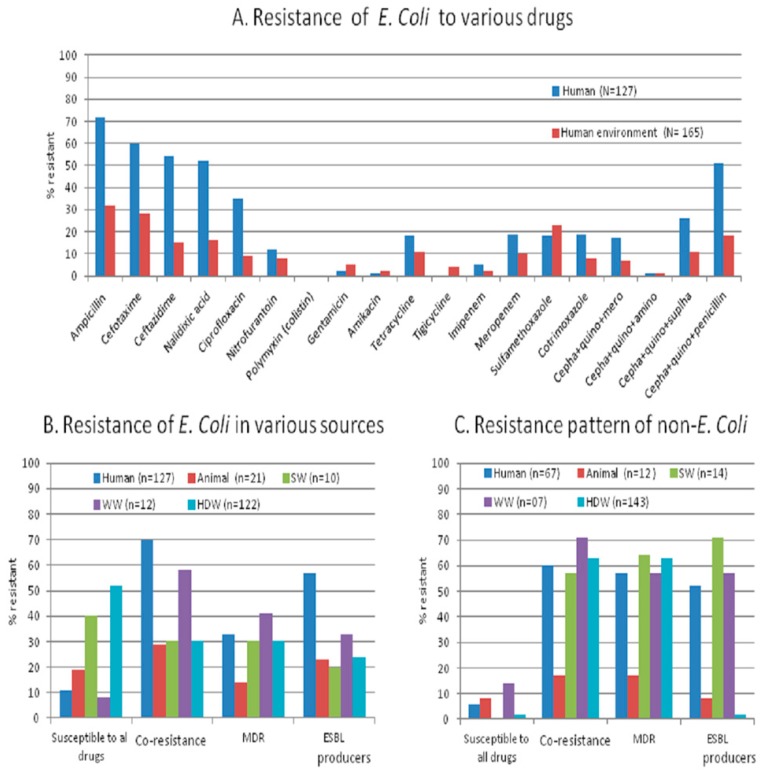
Antibiotic resistance pattern to tested antibiotics in *E. coli* and non-*E. coli* isolates from various sources in a rural setting of Central India. (**A**) Percentage of resistance to various drugs of *E. coli* from human and environmental samples; (**B**) Pattern of resistant *E. coli* isolated from various sources; (**C**) Pattern of resistant non-*E. coli* isolated from various sources. SW: source-water; WW: wastewater; HDW: household drinking water; MDR: multidrug resistance; ESBL: extended spectrum beta-lactamase producers.

All (six) isolates from one child and two household drinking-water samples were susceptible to all drugs and no isolates from any of the samples showed resistance to all antibiotics. The overall percentage of resistant isolate is significantly higher in samples from humans compared to those from the environment (*p* = 0.04). The percentage of resistance for individual antibiotics is also high in humans, except for gentamycin, amikacin, and tigecycline (Figure 2A). Nearly 70% of human stool had co-resistance *E. coli*, of which 57% (73/127) were extended spectrum beta-lactamase (ESBL) producers, and 33% were MDR isolates. In animals, 19% isolates were fully susceptible, 29% co-resistant, 23% ESBL producers and 14% MDR. Co-resistance was more frequent (MDR 41% and ESBL producer 33%) in wastewater isolates than in source water and household drinking water (MDR in 30% and ESBL producer 24%) isolates (Figure 2B). The load of resistant isolates (described as <3 or ≥3 resistant isolates among six collected *E. coli* isolates per sample), in each sample is significantly higher (*p* = 0.001) in human stool than in household drinking-water samples (Table 3), but the resistant isolates from drinking-water were distributed in a higher number of samples. The samples from nuclear families significantly showed less resistance (*p* = 0.05) than in samples from joint families. The resistance pattern of a child and his/her respective household drinking water was not significantly (*p* = 0.05) dissimilar.

### 3.3. Antibiotic Resistance Pattern of E. coli to Various Antibiotic Groups

There was no resistance to the polymyxin (colistin) group in any of the sample types. There was high resistance frequency to penicillins, quniolones, and cephalosporins in human (23%–77%) and environmental (12%–25%) isolates. The MDR combinations having penicillin + cephalosporins + quinolones and sulfonamides + cephalosporin + quinolones groups of drugs were more common than the cephalosporin + quinolone + aminoglycosides or carbapenem combinations (Figure 2A). Most of the isolates from all the sources showed resistance simultaneously to ceftazidime, cefotaxime, cefapime, ampicillin, tetracycline, and co-trimoxazole.

### 3.4. Antibiotic Resistant Genes in E. coli

In human stool, plasmid-mediated cephalosporin-coding genes of the *bla_CTX-M-1_* group was predominant, especially the gene *bla_CTX-M-1_*. In environmental samples *bla_CTX-M-1_* and *bla_CTX-M-9_* genes were also common (Table 4). Plasmid-mediated quinolone resistance genes (i.e., *qnrA, qnrS*, *qnrS*) were detected in 34% human and in 9% environmental quinolone resistant isolates. The *qnrS* gene was most common in human (23/72), and only three household drinking waters were carrying quinolone-resistant genes from environmental isolates (*n* = 33) (Table 4). The coexistence of *bla_CTX-M-1_* and *qnrS* genes were also common (*n* = 12). Carbapenemases encoding genes *NDM-1*, *VIM*, and *IMP* were not detected in any of the carbapenem resistant isolates (*n* = 35) and colistin-resistant gene *mcr-1* was also not detected in any of the isolates (*n* = 292).

The majority (56%–100%) of cephalosporin, quinolone, and carbapenem resistant *E. coli* isolates belonged to phylogenetic group A and B1 (considered as commensal) and 0%–40% belonged to D (considered as extra-intestinal virulent). Human samples carried significantly higher numbers (30%–52%) of group D isolates than environmental samples (0%–40%) (Table 5). Isolates, which showed susceptibility to all drugs, belonged equally to groups A, B1, B2, and D in human samples, but in environmental samples these isolates mainly belonged to the A or B1 groups. The majority (82%) of isolates carrying resistant genes belonged to phylogenetic group A and B1 and the rest (18%) were categorized into group D. Human stool and wastewater were the source of most of the group D *E. coli* isolates.

### 3.5. Non-E. coli Coliforms and AST Pattern

We have also detected many non-*E coli* coliforms (Table 2). We found higher numbers and types of non-*E. coli* coliforms from water samples than in stool samples (human and animal). The number of suggested total coliforms as grown on HiCrome media in terms of number of *E. coli* (identified as blue-violet colonies) and different non-*E. coli* (identified as different color colonies) CFU/unit volume in MDR-positive water samples is shown in Table 6. Only 6% of non-*E. coli* coliform isolates from human stool were susceptible to all tested drugs, while 57% and 52% isolates were MDR and ESBL producers (Figure 2C), respectively. Animal stool carried lower MDR and ESBL producers as compared to isolates from other sources.

## 4. Discussion

We studied antibiotic resistance and selected antibiotic resistance genes in human stool together with their shared and neighboring environment in a rural community from Central India with a ‘one-health’ approach. We found that the antibiotic resistance pattern and its genetic make-up are essentially the same in commensal bacteria from humans and their environment. The percentage of resistant isolates, including MDR (Figure 1A,B), is higher in humans than in the environment (animal stool and water samples), but the load (number of resistant isolates/sample) is higher in the environment than in humans. The appearance of antibiotic-resistant bacteria in healthy individuals and their environment should be evaluated together to accomplish effective antibiotic resistance control.

The antibiotic resistance profile including certain patterns of co-resistance and MDR (i.e., cephalosporin-quinolone-penicillin, sulphonamide + tetracycline + cephalosporin, quinolones + carbapenem + sulfonamide or + tetracycline) in *E. coli* obtained from humans, animals, source- and household-drinking water are high (57%–69%) in our study area. The presence of co-resistance and MDR signifies that there might be high use of antibiotics inhuman and non-human use in the community. The non-human use of highly-important antibiotics contributes to the resistance against a range of antibiotics [1,2,15]. Van den Bogaard et al. and others have shown that the selective pressure on the commensal microflora due to antibiotic misuse determine the frequency and pattern of resistance in a population [16]. The relatively cheap and commonly prescribed drugs commonly favour high co-resistance [17,18].

We found similar patterns of co-resistance, MDR, and gene carriage in various sources. Nearly 90% of MDR *E. coli* isolates are carrying plasmid-encoded (*bla_CTX-M1_*, *bla_CTX-M9_*, *qnrS*, and *qnrS*) genes, which may indicate the possible spread of the resistance genes between diverse sources. This is similar to another study from India [19]. *_CTX-M_*–producing *E. coli* is the dominant MDR *E. coli* in all parts of Asia and of major clinical significance [20]. The patterns of antibiotic use in the community favor the persistence of plasmids carrying antibiotic resistance genes. The intestine is considered as a 'hot spot' for the transfer of resistance genes between bacteria as the exposure of frequently-used antibiotics to a high density of bacteria favours evolution and dissemination of antibiotic resistance by cell-to-cell contact [21,22]. Additionally, the existing various species of MDR bacteria, as we noticed in MDR non-*E. coli* coliform species, (Table 6, Figure 1C), might also be contributing to the spread of antibiotic resistance genes in the intestine with *E. coli*.

The resistant isolates are distributed in a higher percentage of drinking-water samples compared to human samples. In rural communities, the high level of bacterial contamination is reported in source-water to the extent that it lacks the criteria of safe-water supply for domestic purposes [23]. Studies illustrate that surface water contamination occurs mainly from livestock operations and human sewage and that decreasing livestock access to surface water reduced the fecal coliforms levels by an average of 94% [24]. Treatment processes of water, however, might further result in a selective increase of antibiotic-resistant bacteria and might, therefore, increase the occurrence of multidrug-resistant organisms [11,25]. It has also been observed that the microbiological quality of water in vessels in households is lower than that at the source, suggesting that bacterial contamination is widespread during collection, transport, storage, and drawing of water [26].

In our study, phylogenetic group D (extra-intestinal virulent) *E. coli* isolates with resistant genes are more often found from human stool than from environmental samples (30%–52% vs. 0%–24%). It has been reported that co-location of genes in plasmids not only results in resistance to multiple antibiotics, but also in the increased presence of virulence determinants, which facilitates infections [27]. Indeed, the exposure of commensal bacteria to antibiotics increases the carriage level of resistant organisms that might result in the transmission of resistance to a virulent organism [28]. Johnson et al. [29] reported the horizontal transfer of antibiotic resistance not only between isolates from one source to another, but also from resistant to susceptible isolates in the same source. The number of virulent strains carrying resistant genes in human commensal samples is a matter of public health concern, as it may give rise to infection with an increased risk of treatment failure.

We have not identified any *E. coli* or non-*E. coli* isolates (including all forms of MDR strains) with colistin resistance or *mcr-1* gene carriage. With the emergence of MDR and extensive drug resistant (XDR) strains of Gram-negative bacteria, colistin is considered as one of the few last resort antibacterial agents. Recently, sporadic clinical cases infected with colistin-resistant *E. coli* carrying the *mcr-1* gene has been described in India [30,31]. The plasmid-mediated *mcr-1* gene to colistin resistance is a matter of global alarm as its spread within the human commensal flora could lead to epidemics of virtually untreatable infections. Measures with the ‘one-health’ approach, such as colistin susceptibility testing of MDR isolates from patients, testing of food, animal, environmental isolates, and the reduction of colistin use in food-producing animals would be crucial for effective minimization of *mcr*-*1*-positive commensal dissemination in the community and healthcare facilities.

Our study has some methodological limitations. The study, being from a village, cannot be generalized. There is no reason, however, to believe that the situation in this village is very different from many other villages with similar low socio-economic levels in India. Additionally, in our study, none of the carbapenem-resistant isolates (six imipenem resistant and 29 meropenem resistant isolates) from all sources are carrying any of the tested (*NDM-*1, *VIM*, and *IMP*) carbapenemases encoding genes. Studies showed the presence of *OXA-*48 and *NDM-*1 genes in clinical isolates from India [32,33]. However, in another study from our setting, we did not find any of these genes in either clinical or in hospital waste water [34]. We, however, cannot rule out some different resistance mechanisms in these isolates, which we have not tested. Although our study involves a limited number of animals and sewage water samples, the comparison of multiple types of environmental samples with apparently healthy human samples from community provides us better understanding about the current scenario of antibiotic resistance at the community level. This is required in scientific research for establishing effective measures to mitigate resistance in clinically relevant bacteria.

## 5. Conclusions

We found similar and widespread antibiotic resistance, co-resistance, MDR, and their genetic make-up in commensal bacteria from humans and their environment. The percentage of antibiotic resistance is higher in humans than in the environment, but the load (number of resistant isolates/sample) is higher in the environment than in humans. The study, thus, raises a number of important public health concerns. Firstly, community-based studies should be conducted to quantify attributes of antibiotic resistance to design an effective stewardship program; secondly, there should be a multi-sectorial national alliance with all key stakeholders to discourage non-therapeutic use of antibiotics; and, lastly, a strengthening of antimicrobial policy and antibiotic stewardship in India.

## Figures and Tables

**Table 1:** Socio-demographic characteristics of the families of included children (*n* = 22) in a village in Central India.

Variable	Number (%)
**Family type**	
Nuclear family	6
Joint family	16
**Total number of family members**	162
Male	84/162 (52)
Female	78/162 (48)
**Number of children-**	
**Up to five years of age**	46
Male	25/46 (54)
Female	21/46 (46)
**Between one and three years of age**	24
Male	15
Female	9
**Highest education of family member**	
Primary education (up to 5th grade)	5
Middle	11
Secondary	2
Illiterate	144
**Occupation of head of family**	
Job	1
Farmer	12
Labor/self employed	6
Unemployed	3
**Type of house**	
Kuchcha	11
Pucca/semi-pucca	1/10
**Total number of livestock in all households**	75
**Source of drinking water**	
Piped water into dwelling	1
In-house tube wells/bore hole	1
Hand pump	10
Unprotected dug well	1

Types of house: walls, roof, and floors are made of bamboo, mud, grass, reeds, thatch, plastic/polythene, loosely-packed stone, etc., in Kachcha houses, stones, bricks packed with lime or cement mortar or concrete, in pucca houses, while in Semi-Pucca houses walls and roof are of concrete or un-burnt bricks, but the floor is made of mud or non-concrete items.

**Table 2:** Samples and commensal coliforms isolated from human and animal stool and water samples collected from a village in Central India.

Source of Samples	Number of Samples	Number of *E. coli*	Number of Non-*E. coli*
Children stool	22	127	67
Dog stool	1	6	2
Hen stool	1	6	6
Goat stool	1	3	0
Horse stool	1	6	4
Source-water	2	10	14
Waste-water	2	12	7
Household drinking water	22	122	143
Total	52	292	243

**Table 3:** Distribution of various antibiotic resistant *E. coli* isolates in human and drinking water collected from households in a village in Central India.

Name of Antibiotic Tested	Human Stool (*n* = 22)		Household Drinking Water (*n* = 20)	
	Resistant *E. coli* Isolates in Samples (*n*) *			
	<3	≥3	<3	≥3
Ampicillin	6	12	15	3
Ceftazidime	7	11	13	1
Cefotaxime	7	11	11	-
Nalidixic acid	5	9	6	2
Ciprofloxacin	5	6	7	-
Nitrofurantoin	1	1	2	-
Gentamicin	2	1	2	-
Amikacin	1	-	2	-
Tetracycline	1	3	8	-
Tigicycline	3	1	2	-
Imipenem	-	1	-	-
Meropenem	5	2	1	-
Sulfamethoxazole	2	4	4	2
Cotrimoxazole	2	4	4	1

HDW: household drinking-water; *: *p* = 0.001

**Table 4:** Antibiotic resistant genes in commensal *E. coli* isolated from samples from humans and their shared environment from a village in Central India.

**Cephalosporin Resistant Isolates**	**Cephalosporin Resistance Genes**		
	***CTX-M1***	***CTX-M2***	***CTX-M9***
HS (*n* = 73)	62	0	0
HDW (*n* = 26)	11	0	0
AS (*n* = 6)	4	0	0
SW (*n* = 5)	0	0	1
WW (*n* = 5)	0	0	5
**Quinolone Resistant Isolates**	**Quinolone Resistance Genes**		
	***qnrA***	***qnrS***	***qnrS***
HS (*n* = 72)	0	2	23
HDW (*n* = 13)	0	0	3
AS (*n* = 2)	0	0	0
SW (*n* = 1)	0	0	0
WW (*n* = 8)	0	0	0

HS: human stool; AS: animal stool; SW: source-water; WW: wastewater.

**Table 5:** Phylogenetic grouping of resistant commensal *E. coli* isolates collected from various samples from a village in Central India.

Phylogenetic Group	A *n* = 135	B1 *n* = 55	B2 *n* = 13	D *n* = 92
**Cephalosporin-Resistant Isolates**				
HS (*n* = 73)	35	17	0	21
HDW (*n* = 26)	16	3	0	7
AS (*n* = 6)	4	0	1	1
SW (*n* = 5)	4	0	1	0
WW (*n* = 5)	0	0	0	5
**Quinolone-Resistant Isolates**				
HS (*n* = 72)	28	13	1	30
HDW (*n* = 13)	9	1	0	3
AS (*n* = 2)	2	0	0	0
SW (*n* = 1)	1	0	0	0
WW (*n* = 8)	1	0	0	7
**Meropenem-Resistant Isolates**				
HS (*n* = 19)	10	1	0	8
HDW (*n* = 8)	6	2	0	0
AS (*n* = 1)	1	0	0	0
SW (*n* = 1)	1	0	0	0
WW (*n* = 0)	0	0	0	0
**Susceptible to All Drugs**				
HS (*n* = 21)	2	8	6	5
HDW (*n* = 9)	1	6	1	1
AS (*n* = 2)	0	2	0	0
SW (*n* = 3)	1	0	2	0
WW (*n* = 1)	0	1	0	0

**Table 6:** Load of commensal non-*E. coli* and *E. coli* isolates from various water samples carrying multi-drug resistant *E. coli.*

Sample *	Total Coliform Count/100 mL	Total *E. coli*/100 mL *n* (%)	Total-*E. coli* = Non-*E. coli n* (%) (Calculated)
1	1630	260 (16)	1370 (84)
2	1400	40 (3)	1360 (97)
3	520	100 (19)	400 (81)
4	498	64 (13)	434 (87)
5	430	40 (8)	390 (92)
6	414	14 (3)	400 (97)
7	152	3 (2)	149 (98)
8	150	1 (0.66)	149 (99.4)
9	134	34 (25)	100 (75)
10	48	20 (41)	28 (59)
11	365,000,000	15,000,000 (41)	215,000,000 (59)
12	204,000,000	32,000,000 (16)	1,720,000,000 (84)
13	3650	150 (4)	3500 (96)
14	3	0	3 (100)

*: The samples 1–10 are from household drinking water, 11–12 from village waste-water, and 13–14 are from source drinking water.
